# Education as an effective strategy to promote nutritional knowledge, attitudes, and behaviors in street children

**DOI:** 10.1186/s12889-023-15400-9

**Published:** 2023-05-27

**Authors:** Zahra Arabbadvi, Zohreh Khoshnood, Golnaz Foroughameri, Mahboobeh Mazallahi

**Affiliations:** 1 Razi Faculty of Nursing and Midwifery, Kerman University of Medical Sciences, Kerman, Iran; 2 Nursing Research Center, Kerman University of Medical Sciences, Kerman, Iran; 3 Department of Community Health Nursing, Razi Faculty of Nursing and Midwifery, Kerman University of Medical Sciences, Kerman, 7619813555 Iran; 4 Department of Critical Care Nursing, Razi Faculty of Nursing and Midwifery, Kerman University of Medical Sciences, Kerman, Iran

**Keywords:** Nutrition Education, Nutritional Knowledge, Attitudes, Behaviors, Street Children

## Abstract

**Background:**

Lack of nutritional knowledge and ineffective attitudes can complicate the problems faced by this group of street children and have significant effects on their behaviors. This study aimed to examine the effect of nutrition education on nutritional knowledge, attitudes, and behaviors of street children in Kerman in 2021.

**Methods:**

This experimental study was conducted on 70 street children supported by Aftab Children Support Center in Kerman in 2021. The participants were selected using convenience sampling and were divided into two intervention and control groups using a random number table. A nutrition distance education program was implemented using an educational compact disk (CD) for the participants in the intervention group, while the children in the control group did not receive any training. The children’s nutritional knowledge, attitudes, and behaviors were assessed before and one month after the intervention using the Nutritional Behavior Questionnaire. The collected data were analyzed with SPSS software (version 22) using the chi-square test, paired and independent samples t-test, and analysis of covariance (ANCOVA).

**Results:**

The results revealed a significant difference in nutritional knowledge, attitudes, and behaviors after the intervention (*p* < 0.001) due to the effect of the nutrition training program. Accordingly, the mean scores of the participants in the intervention group for nutritional knowledge, attitudes, and behaviors increased by 11.45, 14.80, and 6.05 units after the intervention compared to their scores before the intervention. Furthermore, the effects of the training program on the participants’ nutritional knowledge, attitudes, and behaviors were 89.6%, 91.5%, and 64.3%, respectively.

**Conclusion:**

The findings of this study concluded that training based on nutrition education improved the children’s nutritional knowledge, attitudes, and behaviors. Thus, the officials in charge of promoting the health of vulnerable groups in the community need to provide the necessary facilities to implement effective training programs for street children and encourage them to participate in training programs.

## Introduction

The issue of street children has been one of the most important social problems in recent decades. The presence and activity of children on the streets is a phenomenon that violates cultural, family, and collective values in the world [1]. In 2017, UNESCO estimated that there were about 150 million street children in the world [2]. UNICEF estimated that there are tens of millions of street children worldwide, 90 percent of whom live in low and middle-income countries. There are 150,000 street children in Ethiopia, more than 300,000 street children in Kenya, more than 1,000,000 in Egypt, more than 400,000 in Bangladesh, more than 7 million in Latin America, 500,000 in the six most populous cities of India, more than 16,000 children in Russia working and living on the streets of cities [3, 4]. In a study in 2018, the number of street children in six major cities of Iran was estimated at 26,000 [5].

According to UNICEF, there are three groups of street children: Children living alone on the streets, children who come to work from home, and children who live on the streets with their families. Although these children are different in many ways, they all have one thing in common. They spend most of their time on the streets and are deprived of their basic rights of education, health, nutrition, and security as they spend long hours on the streets and experience many problems [6, 7]. In general, economic, sociocultural, family, and individual factors play a role in the emergence of the phenomenon of street children [7].

The lifestyle adopted by homeless children makes them more vulnerable to health risks and problems than children living at home. These children have poor health due to factors such as homelessness, high-risk sexual behaviors, substance abuse, and violence, and are exposed to physical injuries, violence, sexual abuse, mental health issues, and reproductive health disorders [8]. Street children do not have adequate shelter, proper health and nutrition, and health care. Serious health problems experienced by street children affect their conditions in both childhood and adulthood [6].

A child’s nutrition affects not only their health but also their growth and development. The main goal of child health programs is to promote children’s nutrition and health [9]. Chronic malnutrition in childhood is associated with slower cognitive development and serious future health disorders that reduce the quality of life. Nutritional status is one of the important indicators of quality of life [10]. Street children often work in unfavorable conditions that are dangerous for their health, and many of them are reported to suffer from hunger [8]. The poor health of street children is a chronic problem for them. They are underweight and have poor growth [11]. Not only are street children underweight, but their growth is often stunted, and many of them suffer from chronic and systemic diseases due to nutritional poverty and poor health [12].

Knowledge and attitude are important factors that affect diet. Nutritional knowledge may directly affect diet or indirectly through nutritional attitudes. Nutritional behaviors may become dietary patterns and affect food consumption [13]. Nutritional behavior is defined as the thoughts, actions, and tendencies a living being exhibits to introduce solid and liquid substances into their body [14]. Children’s nutritional behaviors and nutritional knowledge can be improved through education. Although nutritional education does not always directly affect individuals’ nutritional behaviors, it can significantly affect their attitudes, intentions, and thoughts [15].

A study by Cown et al. (2017) in Spain showed that there was no significant difference between the knowledge scores of Hispanic children in the intervention and control groups before the intervention, but there was a significant difference between the knowledge scores of the children in the intervention and control groups after the educational intervention [16]. Ebrahimi et al. (2016) found that the educational intervention induced a positive and increasing change in knowledge, attitudes, and especially healthy eating behaviors of students in the intervention group [17]. A 2013 study conducted by Vakili et al. on middle school students in Yazd revealed that the nutrition education program on milk and dairy consumption improved the students’ knowledge, attitude, and nutritional behavior [18].

Educational programs for street children play a vital role in increasing children’s knowledge, improving their attitudes, and empowering them in self-care. Moreover, given the significant role of community health nurses in providing preventive healthcare services, health education, and health promotion in the community, especially for vulnerable groups, it is essential to implement educational programs for street children. In Iran, particularly in Kerman, only a few studies on the nutrition and education of street children have been conducted, and according to the team’s exhaustive search, limited studies that did not comprehensively and accurately address this issue were found in this field. Therefore, the current study was conducted to determine the impact of proper nutrition education on Kerman’s street children’s knowledge, attitude, and nutritional behaviors in 2021.

## Materials and methods

This experimental intervention-educational study was conducted at Aftab Street Children Support Center in Kerman, affiliated with Kerman Welfare Organization. The research population included 200 street children supported by the center. After the confirmation of the research protocol and obtaining the necessary permits and informed written consent from the children, the participants were selected using convenience sampling. According to the inclusion criteria, children who lacked the necessary equipment at home to view educational videos were excluded from the study. Hence, the remaining 70 street children were placed into the intervention and control groups (each with 35 children) using a random number table. The inclusion criteria were being aged 6–18 years, receiving support from the center, having no specific mental and physical illness (based on the information recorded in the child’s file), having the ability to understand and speak Persian, and having the facilities needed to watch educational videos about healthy nutrition. The exclusion criterion was the child’s failure to complete the training intervention for any reason. The educational content of the principles of proper nutrition was delivered to the center’s psychologist in the form of an educational CD containing ten videos and animations, totaling 120 min. The children were sent to the educational support center after being assigned to the intervention group. The psychologist and the center’s director referred to and explained to the families of Aftab children how to view and use educational content at home regarding the order and titles of the content on three consecutive days.

Following G*Power 3.1 requirements effect size d = 0.7, α = 0.05, and β = 0.80 (and the results of a pilot study on nutritional knowledge), the minimum sample size was estimated as 70 persons.

The data in this study were collected using the Nutritional Behavior Questionnaire developed by Shahmohammadi et al. (2016) [19]. The questionnaire has four sections that assess respondents’ demographic characteristics and nutritional knowledge, attitudes, and nutritional behaviors. The first section contains 13 items that measure demographic characteristics, including gender, age, nationality, weight, height, education, family size, father’s job, mother’s job, residence, birth order, and the number of siblings. In the Aftab street children’s support center, the psychologist measured height in meters and weight on scales. Weight was in kilograms and height in centimeters, and they were then standardized for better comprehension. The second section of the questionnaire contains 16 items that measure nutritional knowledge. This section comprises a total score but no component scores. Each question has a score between 1 and 3, including whether excessive consumption of sweets causes tooth decay and whether milk and dairy products are beneficial for strengthening teeth and bones in humans. The items answered correctly are scored 1, the items answered with no idea are scored 2, and the items answered correctly are scored 3. A respondent’s total score ranges from 16 to 48. The third section of the questionnaire assesses nutritional attitudes using 12 items on a 5-point Likert scale (1 = strongly disagree, 2 = disagree, 3 = neither agree nor disagree, 4 = agree, and 5 = strongly agree) with the minimum and maximum scores of 12 and 60. This section has a total score but no component scores. Each question carries a score between 1 and 5 and consists of questions such as “In my opinion, fruit is not considered food, and daily consumption isn’t required” and “ if I want to remain healthy, I must drink milk daily.” Finally, the fourth section of the questionnaire measures nutritional behaviors (9 items) on a four-point scale, with the higher score given to the correct behavior and the lowest score assigned to the most incorrect behavior. A respondent’s maximum and minimum scores are 9 and 36, respectively. This section comprises a total score but no component scores and includes questions such as, “how many days per week do you typically consume fruit?” and “how many days per week do you usually eat bread, cheese, eggs, milk, and nuts?”.

The validity of the instrument was assessed using face and content validity and a panel of experts. The content validity ratio (CVR) calculated for the whole questionnaire was equal to 0.87, and the content validity index (CVI) was equal to 0.85. In addition, the impact score for face validity was calculated to be 2.87. The reliability of the questionnaire was also assessed using the test–retest method with a Pearson correlation of 0.80. The internal consistency of the questionnaire was estimated using Cronbach’s alpha, and the corresponding value was equal to 0.82 [19].

The data were collected by administering the questionnaire to the children in both intervention and control groups before and one month after the intervention. The children in the control group only completed the items in the questionnaire at the same time as the participants in the intervention group but did not receive any training. As this study was carried out during the COVID-19 pandemic, the children in the intervention group were educated using a two-hour CD. To this end, the children watched educational movies on the CD at home. The instructional content in the CD focused on the importance of nutrition in life, the principles of nutrition, the concept of the food pyramid, the food groups, dietary guidelines, nutritional needs in school age and adolescence, micronutrients and their role in health, the role of nutrition in preventing common non-communicable diseases, the food pyramid and its groups, healthy foods, and the instructions on food labels.

The protocol for this study was approved with the code of ethics IR.KMU.REC.1400.166 by the Ethics Committee of Kerman University of Medical Sciences. The researcher obtained a permit to attend the Street Child Support Center from the Welfare Organization of Kerman Province. To comply with ethical considerations, the children were free to participate in the study or leave it at any stage if they wished. They were also assured about the confidentiality of their information. Besides, the CDs containing healthy nutrition instructions were given to the children in the control group at the end of the study. Before conducting the study, the researchers provided some information to the participants about the objectives of the study, the intervention procedure, and the freedom to leave the study at any time. Informed written consent was obtained from legal guardian(s), and the officials at the Children Support Center. In addition, a special code was assigned to each participant to keep their information confidential.

The collected data were analyzed using SPSS-22 software. The chi-square test was used to compare the demographic variables between the two groups. The paired samples t-test was run to compare the nutritional knowledge, attitudes, and behaviors in each group before and after the intervention. Moreover, the independent samples t-test was used to compare the nutritional knowledge, attitudes, and behaviors between the two groups before and after the intervention. To assess the effectiveness of the intervention on the dependent variables, analysis of covariance (ANCOVA) was used with the control of the variable before the intervention as a covariate. Before conducting the tests, the Shapiro–Wilk and Loon tests were used to verify the assumptions of normality and homogeneity of variances, and the results confirmed their validity.

## Results

Analysis of the data indicated that most of the participants in the intervention and control groups (60% vs. 60%) were in the age range of 10 to 15 years, had a height of 130 to 160 cm (68.6% vs. 51.4%), were studying at primary school (42.9% vs. 60%), lived with their parents (71.4% vs. 67.7%), and their mothers were housewives (74.3% vs. 82.9%). Furthermore, there was no significant difference between the two groups in terms of demographic characteristics (*P* > 0.05) (Table 1).

**Table 1:** Descriptive statistics of demographic characteristics of street children in Kerman city in two groups (*n* = 70) in 2021

Groups		Intervention		Control		Statistic test	*P*-value
Variables		n	%	N	%		
Gender	Female	23	65.7	17	48.6	2.10^a^	.147
	Male	12	34.3	18	51.4		
Age(year)	< 10	11	31.4	10	28.6	.19^a^	.909
	10–15	21	60	21	60		
	> 15	3	8.6	4	11.4		
Weight (kg)	< 10 kg	3	8.6	5	14.3	1.21^a^	.547
	20–50 kg	28	80	24	68.6		
	> 50 kg	4	11.4	6	17.1		
Height (cm)	< 130 cm	11	31.4	6	17.1	2.42^a^	.298
	130–160 cm	18	51.4	24	68.6		
	> 160 cm	6	17.1	5	14.3		
Education	Illiterate	6	17.1	1	2.9	4.92^a^	.178
	Primary school	15	42.9	21	60		
	Junior high school	11	31.4	9	25.7		
	Senior high school	3	8.6	4	11.4		
Living conditions	Living with the mother	10	28.6	12	34.3	.27^a^	.607
	Living with both parents	25	71.4	23	65.7		
The father’s job	Unemployed	12	34.3	17	48.6	1.71^a^	.425
	Worker	12	34.3	8	22.9		
	Self-employed	11	31.4	10	28.6		
The mother’s job	Housewife	26	74.3	29	82.9	2.95^a^	.230
	Worker	5	14.3	1	2.9		
	Self-employed	4	11.4	5	14.2		

^a^Chi-square test

The results of the independent samples t-test showed no statistically significant difference between the two intervention and control groups in terms of nutritional knowledge (*P* = 0.066), attitudes (*P* = 0.887), and behaviors (*P* = 0.128) before the intervention. Thus, the participants in the two groups were homogeneous in terms of the mentioned variables. Table 2 shows the nutritional knowledge, attitudes, and behaviors of the street children in the two groups before and after the intervention.

**Table 2:** Comparison of nutritional knowledge, attitudes, and behaviors of children in Kerman city in 1400 before and after the implementation of the training program on principles of proper nutrition (*n* = 70)

Variables	Tim-Group	Pre-intervention M ± SD	Post-intervention M ± SD	Intragroup Differences	Paired t-test	*P*- value
Knowledge	Intervention	19.62 ± 1.41	31.05 ± 2.43	11.45	-25.74	** < .001**
	Control	20.25 ± 1.40	19.74 ± 1.46	-.51	1.97	.057
	Independent t-test	-1.86	23.55	23.21		
	*P*-value	.066	** < .001**	** < .001**		
Attitudes	Intervention	23.94 ± 1.69	38.74 ± 2.59	14.80	-25.05	** < .001**
	Control	23.85 ± 3.13	23.00 ± 2.44	-.85	1.96	.058
	Independent t-test	.15	26.11	21.32		
	*P*-value	.887	** < .001**	** < .001**		
Behaviors	Intervention	21.97 ± 1.75	28.02 ± 2.61	6.05	-12.92	** < .001**
	Control	21.11 ± 2.78	21.68 ± 2.73	.57	-1.90	.062
	Independent t-test	1.54	9.92	10.20		
	*P*-value	.128	** < .001**	** < .001**		

Bold *p*-values are significant at the level of ≤ 0.05

The results of the paired samples t-test showed that the intragroup changes in nutritional knowledge (*P* < 0.001), attitudes (*P* < 0.001), and behaviors (*P* < 0.001) in the intervention group were statistically significant, indicating significant increases in the mentioned variables after the intervention. However, there was no significant difference in the mentioned variables in the control group before and after the intervention (*P* > 0.05).

The data from the independent samples t-test showed there was a significant difference between the two intervention and control groups in terms of nutritional knowledge (*P* < 0.001), attitudes (*P* < 0.001), and behaviors (*P* < 0.001) after the intervention, indicating a significantly higher increase in the scores for the mentioned variables in the intervention group compared to the control group.

Analysis of covariance (ANCOVA) was performed to check the results. After controlling the pre-test effect of the research variables, the results showed a significant difference between the two intervention and control groups in terms of nutritional knowledge (*P* < 0.001), attitudes (*P* < 0.001), and behaviors (*P* < 0.001). Moreover, the results indicated that the healthy nutrition training program was effective in improving nutritional knowledge (ƞ^2^ = 0.896), attitudes (ƞ^2^ = 0.915), and behaviors (ƞ^2^ = 0.643) of street children (Table 3).

**Table 3:** The results of covariance analysis for two intervention and control groups in the post-intervention phase (*n* = 70)

		df	Mean square	F	*P*-value^*^	Ƞ^2^
Knowledge	Intercept	1	114.756	29.784	< .001	.896
	Pre-test	1	16.420	4.262	.043	
	Group	1	2214.392	574.718	< .001	
	Error	67	3.853			
Attitudes	Intercept	1	456.875	75.783	< .001	.915
	Pre-test	1	28.760	4.770	.032	
	Group	1	4323.696	717.180	< .001	
	Error	67	6.029			
Behaviors	Intercept	1	77.473	16.762	< .001	.643
	Pre-test	1	176.847	38.263	< .001	
	Group	1	558.908	120.926	< .001	
	Error	67	4.622			

^*^ANCOVA; ƞ^2^: Partial Eta Squared

## Discussion

The present study examined the effect of nutrition education on nutritional knowledge, attitudes, and behaviors of street children in Kerman. The findings indicated that the nutritional knowledge of the street children in the intervention group improved significantly after the intervention compared to the knowledge of the children in the control group, indicating that education has a positive effect on improving the nutritional knowledge of street children. Following this finding, various studies have shown that nutrition education has improved participants’ knowledge. Antwi (2020) reported that nutrition education could positively affect the transfer of nutrition knowledge to primary school children [20]. Likewise, Demiroze et al. (2012) reported that nutritional knowledge significantly improved in the intervention group compared to the control group. Following these findings, it can be acknowledged that teaching nutrition and providing educational materials can induce positive changes in nutritional knowledge [21]. Accordingly, Jia et al. (2011) showed the nutritional knowledge of students in the intervention group was significantly improved after the training intervention [22].data in the present study indicated that the nutritional attitudes of the children in the intervention group improved significantly after the intervention compared to their attitudes before the intervention and also compared to the children in the control group. Thus, it can be argued that educational intervention improved the children’s knowledge and, consequently, their attitudes toward healthy nutrition. Similarly, Harake et al. (2018) reported that, compared to the control group, Syrian refugee children in the intervention group had a significant increase in average daily intake of total energy, dietary fiber, protein, saturated fat, and several key micronutrients. Their findings also confirmed the positive effect of this school-based nutrition intervention on the attitude and nutritional status of Syrian refugee children [23]. Martony (2019) also reported that the nutritional attitudes of the participants in the intervention group improved significantly compared to the nutritional attitudes of the participants in the control group after the training intervention [24].

The present study’s findings showed that the nutritional behaviors of the street children in the intervention group improved significantly after the intervention compared to their behaviors before the intervention and also compared to the children in the control group. Consistent with the data in this study, Chung et al. (2018) found that the adolescents in the intervention group obtained higher scores on dietary recommendations and food choices and showed a significant improvement in receiving all macronutrients and micronutrients [25]. Likewise, Demirozu et al. (2012) found sustained and significant changes in children’s eating behaviors in the intervention group compared to the control group, confirming that education positively changed the children’s eating behaviors [21]. Moreover, Dorado et al. (2015) also reported that the nutritional behaviors of Filipino schoolchildren in the intervention group improved significantly compared to the children in the control group [26].

Since the present study was conducted during the Covid-19 pandemic, it was impossible to hold face-to-face training sessions for the children. Besides, given the poor financial ability of street children, most did not have the facilities to watch educational videos at home and, as a result, were excluded from the study. Some of these children were illiterate due to economic poverty and needed help completing the questionnaire; To solve this problem, the items in the questionnaire were read for the children, and the questionnaires were completed for them by a psychologist. Some children were also unwilling to participate in the study. Thus, the researcher gave them food and gifts to encourage them. If the COVID-19 pandemic disappears, face-to-face training programs can be held for children, and compare the results with the findings of this study.

## Conclusion

The findings of this study showed that implementing educational interventions for street children can be a successful strategy to improve their nutritional knowledge and attitudes and correct their eating behaviors. The results of this study also indicated that improving children’s nutritional knowledge and attitudes and correcting their eating behaviors can be an effective steps to reducing the vulnerability of these children. Thus, nurses, especially community health nurses, can play an effective role in designing and implementing educational programs for vulnerable groups in the community, including street children. Therefore, the necessary facilities must be made available to implement street children’s training programs. Furthermore, community health nurses also need to collaborate with street children protection centers to identify these children, formulate guidelines and regulations, and conclude agreements with these centers to help them fulfill their social responsibility. Adopting this approach can contribute to changing the incorrect behaviors of street children and maintaining their health. Following the findings of this study, future studies can examine the nutritional health literacy of street children and explore the effect of educational interventions on the knowledge, attitudes, and behaviors of this group of children. Due to the turbulent economic situation and lack of responsible parents, we cannot improve these individuals’ behavior. However, support organizations such as Welfare can be very helpful in this regard, as well as raising awareness, and it is most often due to a lack of awareness and attitude that there are deficiencies in feeding these children. This education transforms the foods these individuals purchase into healthy foods.

## Data Availability

All data generated or analyzed during this study are included in this published article.
